# Masticatory Loading and Ossification of the Mandibular Symphysis during Anthropoid Origins

**DOI:** 10.1038/s41598-020-62025-8

**Published:** 2020-04-06

**Authors:** Matthew J. Ravosa, Christopher J. Vinyard

**Affiliations:** 1 Departments of Biological Sciences, Aerospace and Mechanical Engineering, and Anthropology, University of Notre Dame, Notre Dame, Indiana 46556 USA; 2 Department of Anatomy and Neurobiology, Northeast Ohio Medical University, Rootstown, Ohio 44272 USA

**Keywords:** Biological anthropology, Biomechanics

## Abstract

An ossified or ‘fused’ mandibular symphysis characterizes the origins of the Anthropoidea, a primate suborder that includes humans. Longstanding debate about the adaptive significance of variation in this jaw joint centers on whether a bony symphysis is stronger than an unfused one spanned by cartilage and ligaments. To provide essential information regarding mechanical performance, intact adult symphyses from representative primates and scandentians were loaded *ex vivo* to simulate stresses during biting and chewing – dorsoventral (DV) shear and lateral transverse bending (‘wishboning’). The anthropoid symphysis requires significantly more force to induce structural failure vs. strepsirrhines and scandentians with unfused joints. In wishboning, symphyseal breakage always occurs at the midline in taxa with unfused conditions, further indicating that an ossified symphysis is stronger than an unfused joint. Greater non-midline fractures among anthropoids suggest that fusion imposes unique constraints on masticatory function elsewhere along the mandible, a phenomenon likely to characterize the evolution of fusion and jaw form throughout Mammalia.

## Introduction

**Figure 1:**
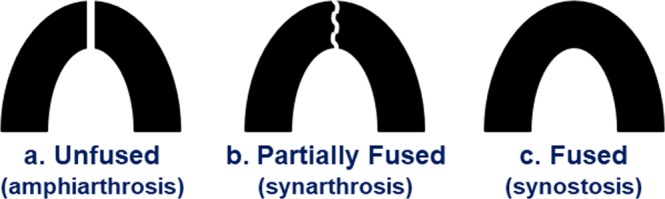
Variation in symphyseal character states across adult primates. Superior views of the symphysis in primitive (**a**: unfused), intermediate (**b**: partially fused) and derived (**c**: fused) conditions. The former two conditions are observed in extant strepsirrhines (Table 1), while modern anthropoids are characterized by only complete fusion of the mandibular symphysis.

The mandibular symphysis represents the third jaw joint of the mammalian masticatory system. It is one of the most interesting and morphologically complex joints in the body, varying from the primitive condition of smooth, opposing dentaries loosely connected by fibrous tissue, ligaments, a fibrocartilage pad and neurovascular bundles (amphiarthrosis) to a more tightly bound joint with greater sutural complexity consisting of variably interlocking rugosities and variably calcified ligaments (synarthrosis) to the derived condition of a wholly ossified or fused joint (synostosis) (Fig. 1)^1–7^. This remarkable evolutionary diversity in symphyseal character states is witnessed during the origin of Anthropoidea, where stem groups exhibit an unfused joint while crown clades such as Catarrhini and Platyrrhini evolved the synapomorphic condition of early ontogenetic fusion^8–15^. Symphyseal fusion has likewise characterized the evolution of diverse mammals, including extinct strepsirrhine primates as well as marsupials, megachiropterans, carnivorans, proboscideans, hyracoids, sirenians, artiodactyls and perissodactyls^2–7,16–22^.

Despite considerable experimental and comparative research, the functional significance of symphyseal fusion remains disputed, being linked either to joint strength or joint stiffness. This relates to the dual potential benefits and consequences of a joint formed by cortical bone. On one hand, a simple, unfused symphysis can experience considerable independent movement between mandibular halves – rotation about three axes and translation in at least two directions^4,23,24^. Taxa with intermediate levels of fusion experience reduced independent jaw movements depending on the orientation, number and mechanical properties of the cruciate and transverse ligaments as well as the degree of interdigitation among bony rugosities (Fig. 1)^2–4,25^. The apomorphic condition of a fused joint facilitates synchronous movements of the left and right jaws due to jaw-muscle activity on a single side^26–28^. It has been argued that ossification stiffens the symphysis for the effective transfer of transversely-oriented balancing-side (BS) jaw-adductor muscle forces during unilateral postcanine chewing and biting^29,30^ or vertically-oriented BS forces during off-midline incisal biting^31,32^. Such ‘Stiffness Models’ posit there is no necessary correspondence between symphyseal fusion and joint strength, with an unfused configuration being just as strong as an ossified one.

**Figure 2:**
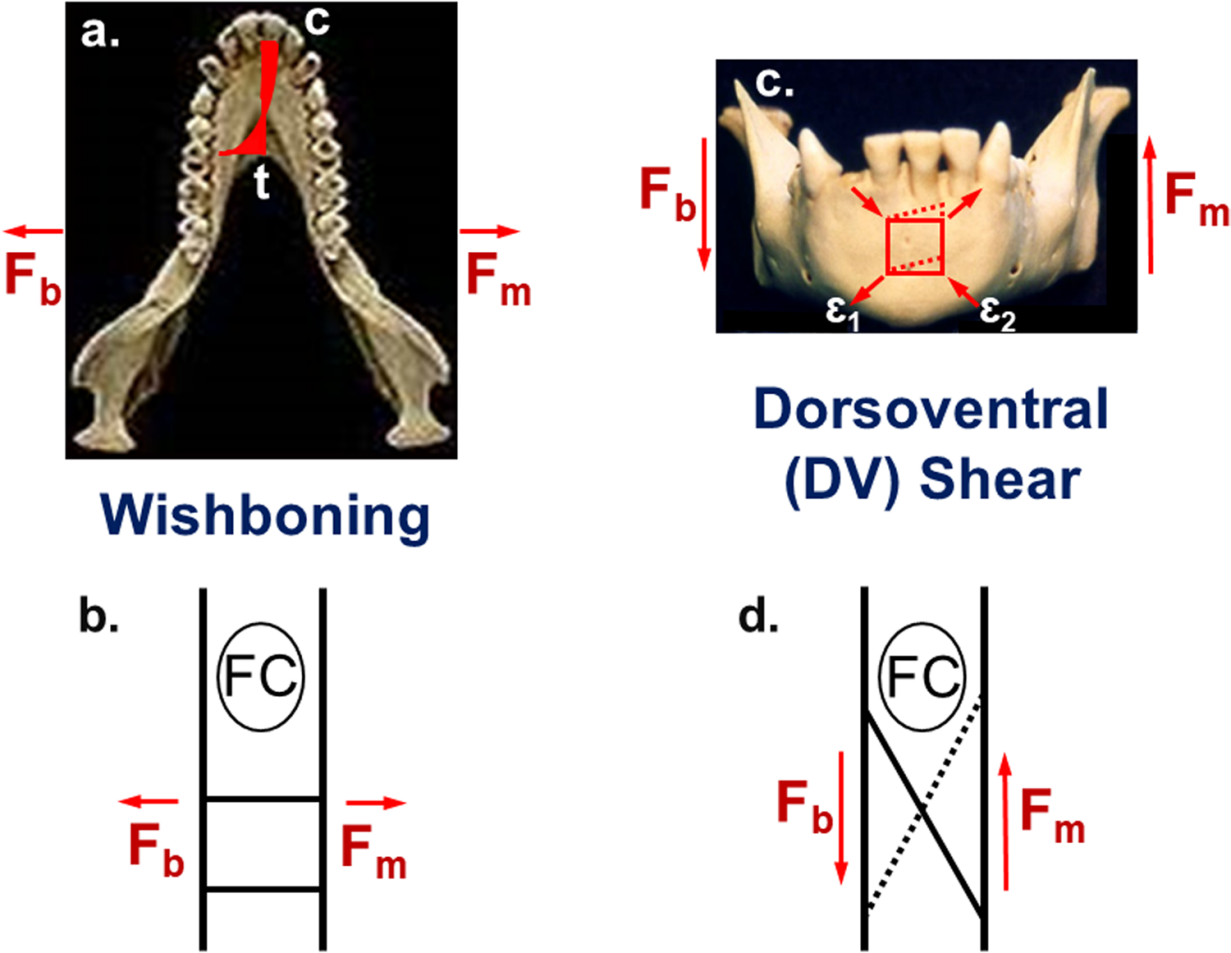
Symphyseal loading patterns during unilateral mastication. Superior views (**a**,**b**): Wishboning in the transverse plane is due to a laterally-directed component of bite force (F_b_) on the working side and an oppositely-directed jaw-adductor force (F_m_) on the balancing side at terminal phase I of the masticatory power stroke. In 2a, the red shaded area indicates the stress distribution across the joint whereby wishboning results in tension (t) along the lingual side and compression (**c**) along the labial side. Note that the neutral axis where the stresses shift between compressive and tensile is located closer to the lingual border, which results in high stress concentrations lingually due to the steep strain gradient related to the bending of curved beams. (**b**) Depicts an unfused joint with transverse ligaments (two horizontal lines) posterior to the fibrocartilage (FC) pad oriented to resist limited wishboning where the symphyseal surfaces are pulled apart along the lingual aspect and the FC pad is compressed anteriorly. Frontal views (**c**,**d**): DV shear in the coronal plane is due to an inferiorly-directed component of bite force (F_b_) along the working side and a superiorly-directed balancing-side jaw-adductor force (F_m_) along the opposite jaw. In 2c, the dotted rectangle shows how a solid square is deformed under DV shear, where the maximum principal strain (maximum tension) is ɛ_1_ and the minimum principal strain (maximum compression) is ɛ_2_. (**d**) Illustrates an unfused joint where only half of the DV cruciate ligaments (dotted line) are oriented to resist DV shear during *left-sided* chewing. The other cruciate ligaments experience compression and have no role in resisting left-sided DV shear. When chewing is *right-sided*, directions of force resultants are reversed and the cruciate ligaments (oblique solid line) oriented in the opposite direction then experience tension due to DV shear. Increased stiffness of cruciate ligaments due to greater calcification allows such tissues to counter compression and effectively double the amount of material able to resist DV shear. DV shear also results in shear of the labially located FC pad.

On the other hand, anthropoids differ from strepsirrhines with unfused joints in exhibiting higher levels of BS jaw-adductor muscle activity during unilateral mastication, which results in elevated peak strains along the BS mandible and increased dorsoventral (DV) shear and lateral transverse bending (‘wishboning’) of the symphysis (Fig. 2)^6,26–28,33–40^. Mammals with partial or intermediate fusion possess cruciate ligaments spanning the symphysis oriented primarily to resist DV shear; such ligaments are more calcified and attached to variably interlocking bony rugosities (Fig. 1 and 2)^2–4^. A subset of taxa with partial fusion also exhibit transversely-oriented ligaments spanning the posterior joint that appear well-designed to counter moderate levels of symphyseal wishboning (Fig. 2)^2,3,6^. It follows from this *in vivo* and comparative research that variation in joint form and fusion is continuous and potentially proportional to the amount of stress experienced along the symphysis during biting and chewing^2,3,5,6,13,17,26–28,33–39^. Consequently, such ‘Strength Models’ argue that increased fusion strengthens the symphysis against elevated stresses because the cortical bone in a synostosis is stronger than the ligaments and fibrocartilage spanning a similarly sized amphiarthrosis. In this scenario, increased joint stiffness is a by-product of selection for the greater strength conferred by a bony symphysis.

Although morphological transformations in symphyseal fusion are well documented during anthropoid origins^8–15^, surprisingly little data exist to address whether these phylogenetic changes are functionally related to joint strength *or* joint stiffness. This is due, in part, to the paucity of information regarding the mechanical properties of cranial tissues, and because the symphysis is comprised of fibrocartilage and not hyaline cartilage as observed in the postcranium^41^. Indeed, many aspects of the biomechanics of syndesmoses (cranial sutures) and synovial joints (temporomandibular joint) are better documented than for arthroses like the mandibular symphysis^42–45^. To this end, we performed experiments to evaluate the mechanical strength and integrity of the mandibular symphysis in a diverse sample of adult primates. We compared anthropoids and strepsirrhines varying in the degree of symphyseal fusion via *ex vivo* macroscale tests of joint strength during simulated masticatory loads – DV shear and wishboning (Fig. 2; Table 1)^6,26–28,33–39^. In addition to furnishing novel data on symphyseal fusion and joint performance concerning a long-recognized synapomorphy of crown anthropoids, our analyses are highly relevant to investigations that require functional information about jaw form for inferring the behavior of fossil remains.

**Table 1:** Symphysis sample composition for primate suborders and scandentians. Major clade totals are in bold, with species totals noted first and specimen totals listed second.

TAXON	SPECIES/SAMPLE	FUSION
**Primates**	**34/132**	**U, P, F**
**Strepsirrhini**	**20/68**	**U, P**
Cheirogaleidae	4/16	U
Lemuridae	7/25	U, P
Indriidae	2/8	P
Lorisidae	4/10	U
Galagidae	3/9	U
**Anthropoidea**	**14/64**	**F**
Callitrichidae	4/25	F
Cebidae	5/22	F
Cercopithecidae	4/12	F
Hominidae	1/5	F
**Scandentia**	**1/3**	**U**
Tupaiidae	1/3	U

(Key: U = unfused, P = partially fused, F = fused).

## Results and Discussion

Studies of musculoskeletal biomechanics routinely assume that phenotypic variation tracks differences in performance and, ultimately, fitness^46,47^. In terms of anthropoid origins, critical evidence has been lacking as to the mechanical performance of a fused mandibular symphysis, which hinders attempts to understand adaptive transformations in the fossil record^48^. The strength models predict that anthropoids will exhibit relatively and absolutely stronger symphyses that break at the midline to a lower extent than in strepsirrhines and scandentians. Thus, we investigated symphyseal strength and performance in several ways. Regression lines were compared for simulated *ex vivo* loads (wishboning, DV shear) necessary to induce structural failure of fused symphyses for anthropoids vs. strepsirrhines and scandentians with unfused or partially fused joints. To control for potential suborder variation in relative symphyseal size, fracture strengths were scaled to joint size and compared between anthropoids and strepsirrhines. To evaluate if the soft tissues of unfused joints are weaker in wishboning and DV shear, we compared symphyseal midline fracture frequencies during simulated loads between the anthropoid vs. strepsirrhine plus scandentian samples. To explore the relative importance of the two loading regimes on symphyseal strength, we then compared anthropoid regression lines describing the forces required to induce joint failure in wishboning vs. DV shear.

**Figure 3:**
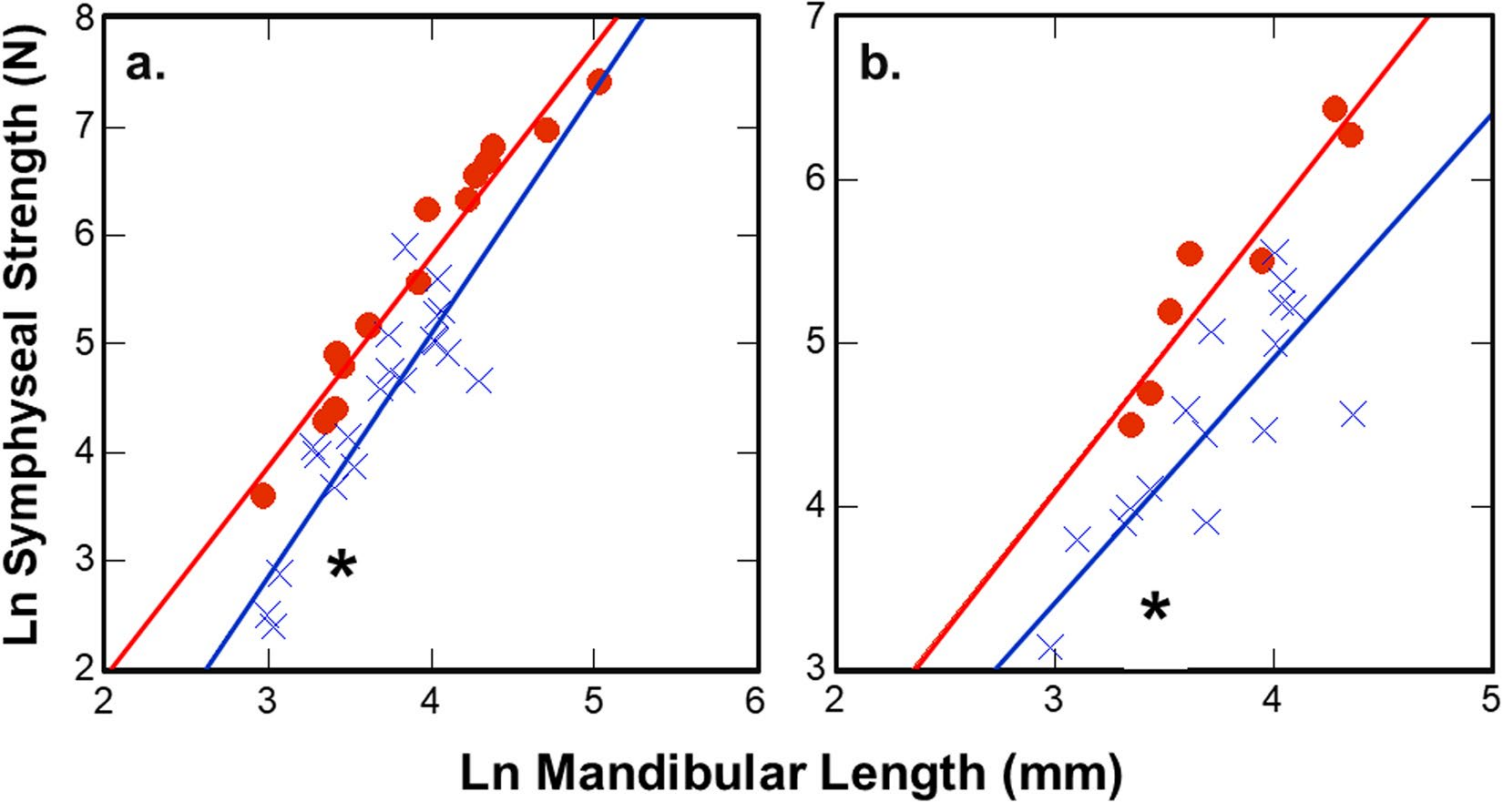
Suborder comparison of symphyseal forces at joint failure during simulated: (**a**) Wishboning in 14 anthropoid, 20 strepsirrhine and 1 scandentian (*Tupaia*) species; and (**b**) DV shear in 7 anthropoid, 16 strepsirrhine and 1 scandentian (*Tupaia*) species. In both cases, the regression line for anthropoids (red) is significantly transposed above that for strepsirrhines and scandentians (blue), indicating that anthropoids have relatively stronger symphyses in lateral transverse bending and DV shear. *Tupaia* (*) exhibits an unfused joint like most strepsirrhines that also functions similarly. (Key:  = Anthropoidea;  = Strepsirrhini; *Tupaia).

Suborder comparisons of the forces required to induce structural failure of the symphysis in wishboning (Fig. 3a) and DV shear (Fig. 3b) indicates that anthropoid allometric trajectories vs. mandibular length are significantly transposed above those for strepsirrhines (Table 2). Accompanying comparisons of symphyseal strength scaled to joint size confirms that anthropoids exhibit significantly higher relative strength in wishboning and DV shear than strepsirrhines (Table 3). In all such analyses, scandentians with unfused joints have similar relative levels of joint strength as strepsirrhines. Therefore, synostosis in anthropoids results in a significantly stronger symphysis, both absolutely and relatively, for countering masticatory stresses than the unfused joint of most strepsirrhines. These results are inconsistent with competing arguments that variation in fusion is unrelated to strengthening the mandibular symphysis^29–32^. Instead, our novel findings regarding symphyseal performance suggest that increased joint stiffness is a secondary consequence of increasing joint strength via synostosis.

**Table 2:** Bivariate regressions and comparisons between and within primate suborders.

Vs. ln Mandibular Length	Strepsirrhini + Scandentia			Anthropoidea^d^		
(‘N’ for suborders)	Y-Intercept^a^	Slope^a^	‘r’^b^	Y-Intercept^a^	Slope^a^	‘r’^b^
ln Wishboning Strength (N = 21/14)^c^	−3.853/−5.154	2.236/2.591 ± 0.300 (CI)	0.863***	−2.005/−2.192	1.953/2.001 ± 0.124 (CI)	0.976***
ln DV Shear Strength (N = 17/7)^c^	−1.079/−2.448	1.497/1.867 ± 0.287 (CI)	0.802***	−0.986/−1.282	1.697/1.794 ± 0.259 (CI)	0.946**

^a^Least-squares regression values are first and reduced major axis values are second.

^b^Significance levels: ***p < 0.0001; **p < 0.001; *p < 0.05.

^c^Anthropoid line significantly up transposed vs. the strepsirrhine line (ANCOVA, p < 0.0001).

^d^Wishboning line not significantly different than DV shear line in anthropoids (ANCOVA, p = 0.747).

**Table 3:** ANOVA comparisons of strepsirrhine and tree shrew vs. anthropoid means for symphyseal strength scaled to joint cross-sectional area (N/mm^2^) and symphyseal midline fracture location frequency during simulated wishboning and DV shear (Midline % Key: 0.000=never midline; 1.000=always midline).

LOADING REGIME VARIABLE	STREPSIRRHINI+SCANDENTIA $\bar{{\rm{x}}}$ (N, SD)	ANTHROPOIDEA $\bar{{\rm{x}}}$ (N, SD)	ANOVA P-Value
Wishboning Stress/Area	2.237 (21, 0.946)	3.012 (14, 0.736)	0.014
DV Shear Stress/Area	2.430 (17, 0.932)	3.538 (7, 1.349)	0.030
Wishboning Midline %	1.000 (21, 0.000)	0.492 (14, 0.460)	0.001
DV Shear Midline %	0.618 (17, 0.485)	0.491 (7, 0.431)	0.467

**Figure 4:**
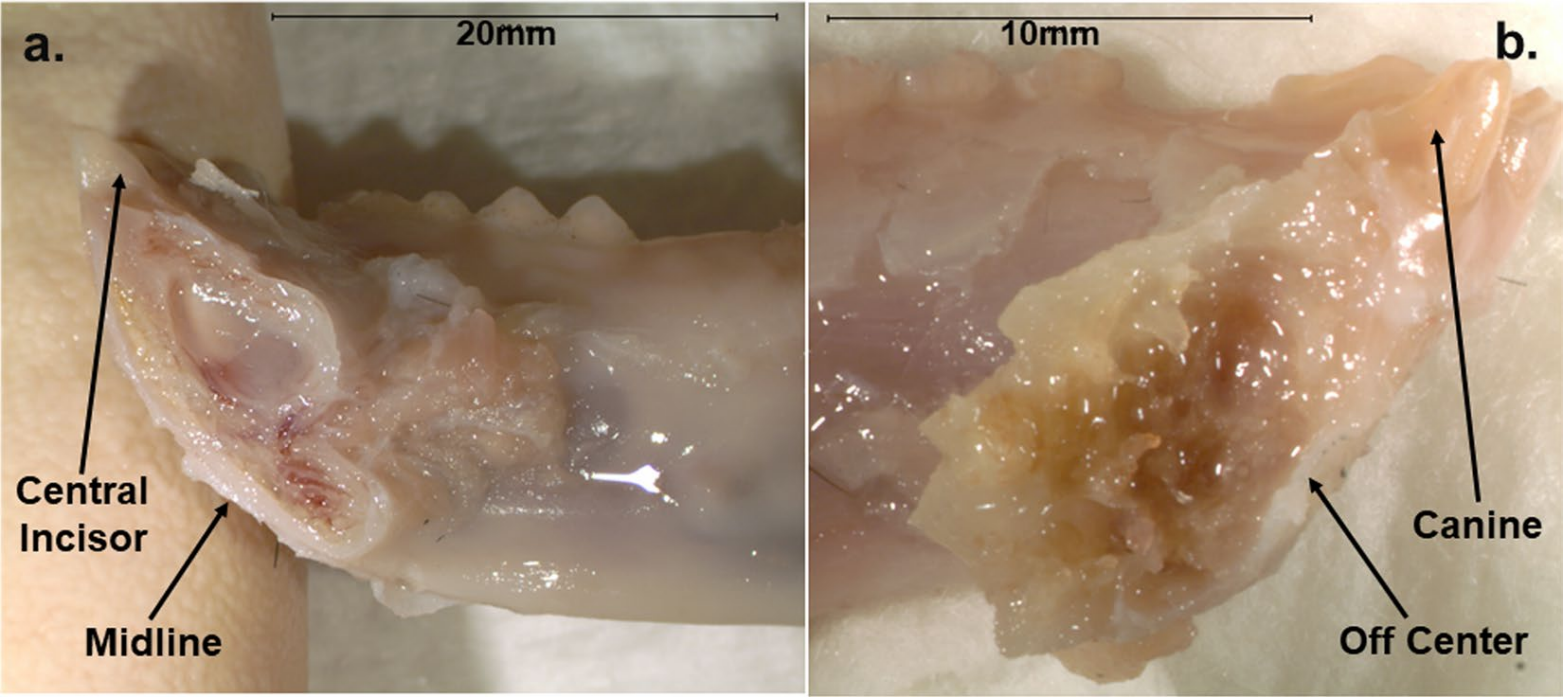
Medial views of different symphyseal fracture patterns during simulated wishboning in primates: (**a**) right mandible and symphyseal surface in a longer-faced macaque with a midline joint fracture between the central incisors; and (**b**) left mandible and majority of the symphysis in a shorter-faced owl monkey with a joint fracture just lateral to the right canine.

**Figure 5:**
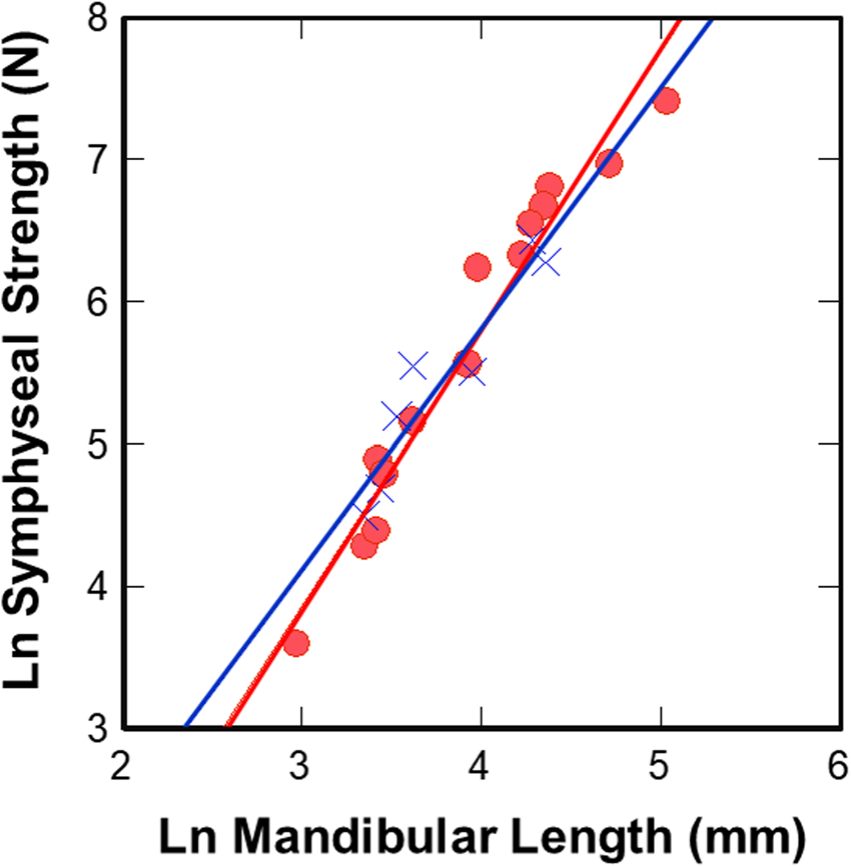
Comparison of symphyseal forces at joint failure during simulated wishboning and DV shear in 14 and 7 anthropoid species, respectively. Regression lines for wishboning (red) and DV shear (blue) in anthropoids are coincidental, suggesting the symphysis is comparably designed for both jaw-loading regimes at a given size. (Key:  = wishboning;  = DV shear).

Comparison of symphyseal failure locations between anthropoids and strepsirrhines indicate that the latter exhibit a significantly higher frequency of midline joint fractures during simulated wishboning loads (Fig. 4; Table 3). This pattern is predicted by the ‘Strength’ models, which posit that symphyseal soft tissues constitute the weakest component of an unfused joint, particularly with regard to high wishboning stress concentrations along the lingual surface of the symphysis where crack propagation is initiated^49,50^. In contrast, there are no suborder differences in the degree of midline joint fractures during DV shear (Table 3). Rather than providing support for the ‘Stiffness’ models, this discrepancy highlights the likelihood that wishboning is the singular functional determinant of symphyseal fusion in anthropoids. Since the anthropoid symphysis is equally strong in countering DV shear as it is in resisting wishboning (Fig. 5; Table 2), this suggests that the greater strength of the anthropoid symphysis in DV shear vs. strepsirrhines is largely due to being designed to resist elevated wishboning. Given that incision has been eliminated as a potential determinant of symphyseal fusion in primates^5,13,17^, our current findings provide unique empirical support for the central role of stresses during postcanine biting and chewing, specifically wishboning, as a determinant of symphyseal fusion in anthropoids.

Indeed, the onset of wishboning and symphyseal fusion imposes a number of unappreciated constraints on phenotypic diversity in the anthropoid masticatory system. Previous work indicates that symphyseal midline fractures are more frequent in marmosets with more curved symphyses than other South American monkeys^49,50^. In our more diverse sample, three prognathic cercopithecine taxa with correspondingly greater joint curvature exhibit 100% midline fractures vs. a mean of 22.6% for nine species of shorter-faced anthropoids (Fig. 4; Supplementary Table 1). Increased symphyseal curvature generates higher wishboning stress concentrations that can be effectively countered via added cortical bone at the joint midline^33,34^. That symphyseal curvature increases with positive allometry during ontogeny and interspecifically in cercopithecine monkeys^34,38^ further underscores the overarching role of wishboning stress on symphyseal function and patterns of mandibular cortical bone distribution during phyletic size change in living and extinct anthropoids. This phenomenon in primate jaws also represents an example of skeletal curvature and load predictability more typically observed in vertebrate limb elements^51^.

As wishboning results in fractures at off-axis locations in more orthognathic anthropoids with less joint curvature, this suggests the presence of structural tradeoffs elsewhere along the mandible that also may be novel for this primate suborder. Herein, non-midline cracks due to wishboning differentially propogated through tooth roots along the anterior portion of the jaw. Many of these cases involved the canine, which is particularly well developed in anthropoids^14^. Although camelids do not exhibit wishboning, the presence of relatively larger incisor roots and less cortical bone along the symphysis has been invoked to explain why this clade is singular among selenodont artiodactyls in having novel functional tradeoffs underlying the evolution of a fused symphysis^21^. Accordingly, the evolution of orthognathy in anthropoids such as hominids may be characterized by greater functional limits on cortical bone quantity and quality in more anterior mandibular sites where large tooth roots represent sources of potential weakness due to the relative reduction of surrounding cortical bone to resist wishboning stress^52,53^.

Additional craniodental features are associated with the evolution of a wishboning loading regime during anthropoid origins. Phase II molar facets are crushing surfaces that are loaded maximally during jaw movement in the latter portion of Phase I occlusion^28,54^. A relatively larger, more transverse component to Phase II molar occlusal facets is noted for early anthropoids^55–59^. In concert with an increased emphasis on postcanine crushing and trends toward isodonty and isognathy^13,60^, the duration of the anthropoid masticatory power stroke occupies a relatively higher percentage of the chewing cycle^61^. Because of these derived occlusal trends, jaw-adductor muscle and jaw-kinematic patterns underscore the greater importance of the transverse component of the masticatory power stroke in anthropoids; they are likely to be linked both functionally and phylogenetically to the evolution of a fused symphysis designed to counter increased wishboning stress.

In sum, our study suggests that the evolution of symphyseal fusion in primates imposes novel constraints on other aspects of masticatory form and function^62^. This is consistent with findings that scandentians with unfused joints exhibit similar levels of joint strength as strepsirrhines. As symphyseal fusion is an important synapomorphy of diverse mammalian clades and does not exhibit evolutionary reversals^13,22^, our data on macroscale joint properties uniquely inform symphyseal trait performance and contribute key insights into the biomechanics of character-state variation and covariation in the skull of living and fossil mammals^46–48,62^.

## Methods

### Sample

Adult primates with unfused (amphiarthrosis), partially fused (synarthrosis) and fully fused (synostosis) symphyses were obtained and intact joints were fixed for mechanical tests of joint strength (Table 1). As an initial test of whether joint form and function is independent of phylogeny, we included a tree shrew species (Scandentia) as an outgroup with an unfused symphysis with the strepsirrhine sample of taxa with unfused and partially fused joints. All primate, tree shrew and rat (below) specimens were obtained postmortem with permission from personal collections, those of colleagues, and the Duke Lemur Center (DLC). Mandibles were used only if the adult dentition was fully erupted and there was a full complement of teeth in functional occlusion. To prevent degradation of soft tissues, joints were fixed in 10% buffered formalin. This facilitated the use of specimens obtained opportunistically due to death from natural causes that needed to be stored prior to experiments as well as frozen specimens from the DLC that uniquely sampled a broad range of rare strepsirrhines. Although fixation increases cross-linking among proteins that may differentially strengthen the cartilage and ligaments of an unfused joint, the mechanical properties of bone and some soft tissues are minimally affected^63–65^. Thus, while the disparity in joint performance among fusion cohorts might be greater in comparisons of ‘fresh’ specimens, the use of fixed specimens greatly increased our total samples. Comparisons of joint strength during wishboning for five adult rat jaws frozen for one month and then fixed vs. five adult rat jaws fixed without freezing indicate no difference between groups (means of 15.4 N vs. 15.8 N, Mann-Whitney U test, p > 0.05). This suggests that variation in sample preparation (frozen then fixed [DLC] vs. fixed only [all other samples]) does not significantly influence variation in the strength of an unfused symphysis vis-à-vis wishboning. If a species sample was comprised of more than one intact symphysis, about half the sample from each taxon was utilized in macroscale tests of joint performance during simulated DV shear. In such cases, the other half was used for macroscale tests of symphysis strength during simulated wishboning. However, due to small sample sizes in a number of taxa, there are differences in species composition for each suborder in the macroscale tests.

### Mechanical tests of joint performance

We determined the strength (i.e., load at failure in Newtons, N) of formalin-fixed, articulated mandibular symphyses from cadavers in 14 anthropoid, 20 strepsirrhine and one scandentian species in either simulated wishboning or DV shear (Table 1; Supplementary Tables 1 and 2). The harvested symphyses were attached to a Universal Testing Machine (Instron, Norwood, MA) and loaded to structural failure in either loading regime at a constant rate of 2.54 cm/min^49,50^. Depending on specimen size, either a 250 N or 5,000 N load cell was used to measure load on each symphysis. To simulate wishboning, we fixed wires or metal posts to both sides of the jaw just posterior to the symphysis (to eliminate/minimize the bending moment arm) and attached the wires/posts to either the load cell or stationary grip. Each symphyseal half was loaded in lateral transverse bending to structural failure. To simulate DV shear, we potted each hemimandible in epoxy and, once hardened, a hole was drilled through the epoxy immediately lateral to the symphysis and orthogonal to the jaw’s long axis. Metal posts were inserted through the hole on each side and oriented in opposite directions prior to being fastened and attached to either the load cell or stationary grip. Subsequently, each symphyseal half was loaded to structural failure in simulated DV shear. For all macroscale tests, we recorded the force required for joint failure in N. While mammals do not load their symphyses to structural failure *in vivo*, predictions regarding *ex vivo* analyses are based on the previously determined correspondence in cortical bone between ultimate failure vs. failure in cyclical loading^66^. For all wishboning tests, fracture location was recorded to determine if ossified joints failed at different locations than in species with unfused joints. Due to a very limited number of taxa with partial fusion in our sample, we combined these force values (N) with those for species with unfused joints.

### Statistics

Least-squares and reduced major axis bivariate regressions (p < 0.05) of species means were performed in each suborder between natural logs of measures of symphyseal strength (N) and mandibular length (0.1 mm). The latter tracks variation in masticatory size and estimates a masticatory load arm. Comparison of least-squares scaling trajectories between suborders employed ANCOVA (p < 0.05); similar analyses were utilized to compare allometric patterns within anthropoids for joint strength in wishboning vs. DV shear. To control for the effect of suborder variation in relative joint size on symphyseal strength, failure stress per joint area (N/mm^2^) was compared between suborders for each loading regime (ANOVA, p < 0.05). In each species, the frequency of specimens where the location of joint failure occurred at the symphyseal midline was calculated. These values were used for comparisons of suborder means performed via ANOVA (p < 0.05). Data used for the bivariate and univariate analyses are located in Supplementary Tables 1 and 2.

## Supplementary information

Dataset 1 and 2.
