# Supplementary material for: Masticatory Loading and Ossification of the Mandibular Symphysis during Anthropoid Origins
Source: Sci Rep. 2020 Apr 6;10:5950. doi: 10.1038/s41598-020-62025-8 (PMC7136211; doi:10.1038/s41598-020-62025-8)
Supplement: Supplementary file 1 — Dataset 1 and 2. [file 41598_2020_62025_MOESM1_ESM.pdf]

Masticatory Loading and Ossification of the Mandibular Symphysis during Anthropoid Origins

Matthew J. Ravosa[1]* and Christopher J. Vinyard[2]

[1]Departments of Biological Sciences, Aerospace and Mechanical Engineering, and Anthropology, University of Notre Dame, Notre Dame, Indiana, 46556

[2]Department of Anatomy and Neurobiology, Northeast Ohio Medical University, Rootstown, Ohio, 44272

*Correspondence to:

Dr. Matthew J. Ravosa, Professor
Department of Biological Sciences
Galvin Life Science Center
University of Notre Dame
Notre Dame, Indiana 46556
Email: Matthew.J.Ravosa.1@nd.edu
Phone: 574-631-6552; fax: 574-631-7413

Co-Author Email: cvinyard@neomed.edu

Supplementary Table 1. Strepsirrhine, Scandentian and Anthropoid Species Means for Wishboning Tests

Species	Family	N	Midline Fracture (%)	Symphysis Strength (N)	Mandible Length (mm)	Symphysis Area (mm^2)	Symphysis Ratio (N/mm^2)
Cheirogaleus major	Cheirogaleidae	1	100	47.690	34.10	28.025	1.702
Cheirogaleus medius	Cheirogaleidae	2	100	57.085	26.50	16.175	3.529
Eulemur coronatus	Lemuridae	2	100	192.46	56.50	49.926	3.855
Eulemur fulvus	Lemuridae	4	100	135.415	60.30	65.603	2.064
Eulemur macaco	Lemuridae	3	100	200.127	58.47	60.037	3.333
Galagoides demidoff	Galagidae	1	100	10.920	20.70	9.499	1.150
Galago moholi	Galagidae	3	100	17.730	21.53	11.919	1.488
Hapalemur alaotrensis	Lemuridae	1	100	361.810	46.50	88.784	4.075
Hapalemur griseus	Lemuridae	2	100	114.230	42.45	63.126	1.810
Lemur catta	Lemuridae	2	100	152.860	55.50	55.920	2.734
Loris tardigradus	Lorisidae	2	100	53.110	27.10	18.362	2.893
Mirza coquereli	Cheirogaleidae	2	100	39.440	30.10	15.963	2.471
Microcebus murinus	Cheirogaleidae	3	100	12.163	19.93	7.607	1.599
Nycticebus coucang	Lorisidae	2	100	160.675	41.95	49.406	3.252
Nycticebus pygmaeus	Lorisidae	1	100	63.160	32.70	31.651	1.996
Otolemur garnetti	Galagidae	3	100	104.936	46.00	48.924	2.145
Perodicticus potto	Lorisidae	1	100	97.970	40.00	52.564	1.864
Propithecus tattersalli	Indriidae	2	100	270.295	56.65	145.068	1.863
Propithecus verreauxi	Indriidae	3	100	149.957	55.87	162.736	0.922
Varecia variegata	Lemuridae	3	100	105.217	73.17	83.249	1.264
Tupaia belangeri	Tupaiidae	2	100	19.174	31.31	19.733	0.972
Alouatta seniculus	Cebidae	1	0	906.600	80.10	302.076	3.001
Aotus trivirgatus	Cebidae	3	0	173.585	37.38	47.145	3.682
Cebus apella	Cebidae	2	50	511.010	53.60	140.604	3.634
Callithrix jacchus	Callitrichidae	7	86	72.437	28.60	34.893	2.076
Cebuella pygmaea	Callitrichidae	2	100	36.290	19.53	12.069	3.007
Pithecia pithecia	Cebidae	3	33	260.380	50.70	89.139	2.921
Saguinus geoffroyi	Callitrichidae	1	100	133.360	30.71	38.277	3.484
Saguinus oedipus	Callitrichidae	4	0	80.823	30.42	41.051	1.969
Saimiri sciureus	Cebidae	5	20	120.019	31.80	37.183	3.228
Macaca fascicularis	Cercopithecidae	3	100	557.119	68.45	176.618	3.154
Macaca mulatta	Cercopithecidae	3	100	788.704	77.40	219.184	3.598
Papio anubis	Cercopithecidae	2	100	1652.045	153.5	1177.390	1.403
Trachypithecus francoisi	Cercopithecidae	2	0	697.940	72.00	173.870	4.014
Homo sapiens	Hominidae	5	0	1057.508	111.50	352.646	2.999

Supplementary Table 2. Strepsirrhine, Scandentian and Anthropoid Species Means for DV Shear Tests

Species	Family	N	Midline Fracture (%)	Symphysis Strength (N)	Mandible Length (mm)	Symphysis Area (mm^2)	Symphysis Ratio (N/mm^2)
Cheirogaleus medius	Cheirogaleidae	2	100	49.101	27.550	14.899	3.300
Eulemur coronatus	Lemuridae	1	0	216.450	56.800	70.462	3.072
Eulemur fulvus	Lemuridae	2	0	183.745	59.600	73.986	2.484
Eulemur macaco	Lemuridae	2	50	189.195	56.600	66.376	2.850
Galago moholi	Galagidae	1	100	44.245	22.200	10.362	4.270
Hapalemur griseus	Lemuridae	1	0	84.480	40.000	50.742	1.665
Lemur catta	Lemuridae	1	100	86.601	52.300	47.493	1.824
Loris tardigradus	Lorisidae	2	0	53.745	28.375	17.875	3.007
Mirza coquereli	Cheirogaleidae	2	100	60.345	31.000	17.619	3.425
Microcebus murinus	Cheirogaleidae	4	100	22.881	19.650	8.2975	2.758
Nycticebus coucang	Lorisidae	1	100	158.710	41.000	51.457	3.084
Nycticebus pygmaeus	Lorisidae	1	100	97.874	36.600	34.540	2.834
Otolemur garnetti	Galagidae	1	100	49.284	40.180	36.088	1.366
Propithecus tattersalli	Indriidae	2	100	147.486	55.100	128.928	1.144
Propithecus coquereli	Indriidae	1	100	257.570	55.000	149.464	1.723
Varecia rubra	Lemuridae	1	0	95.820	78.500	98.580	0.972
Tupaia belangeri	Tupaiidae	1	0	29.427	31.310	19.104	1.540
Alouatta seniculus	Cebidae	1	100	530.730	78.000	215.875	2.459
Aotus trivirgatus	Cebidae	1	0	254.882	37.380	40.722	6.259
Callithrix jacchus	Callitrichidae	7	29	89.448	28.600	32.683	2.737
Pithecia pithecia	Cebidae	1	0	244.920	51.900	91.814	2.668
Saimiri sciureus	Cebidae	5	40	179.294	34.100	41.416	4.329
Saguinus oedipus	Callitrichidae	4	75	108.523	31.125	35.694	3.040
Macaca mulatta	Cercopithecidae	2	100	621.414	72.500	189.829	3.274
